# Dataset on the mechanical property of graphite after molten FLiNaK salt infiltration

**DOI:** 10.1016/j.dib.2018.11.036

**Published:** 2018-11-14

**Authors:** Can Zhang, Hui Tang, Zhoutong He, Jinliang Song, Yantao Gao

**Affiliations:** Key Laboratory of Nuclear Radiation and Nuclear Energy Technology, Chinese Academy of Sciences, Shanghai 201800, PR China

**Keywords:** Graphite, Fluoride molten salt, Infiltration, Mechanical strength

## Abstract

Presented in this article are mechanical property and microstructural data for fluoride molten salt infiltrated graphite at high temperature. Four infiltration pressures (0 kPa, 450 kPa, 600 kPa, and 1000 kPa) and two kinds of graphite (IG-110 and NG-CT-10) were used during molten salt infiltration. After fluoride molten salt infiltration, compression testing and tension testing were performed at 700 °C to determine compressive strength, tensile strength, softening coefficient, stress–strain curve, and absorbed energy. Utilizing scanning electron microscopy (SEM) applied to fracture fragments, SEM micrographs for the fracture surface of molten salt infiltrated graphite and virgin graphite were determined.

**Specifications table**

**Table:** 

Subject area	*Materials science*
More specific subject area	*Nuclear material*
Type of data	*Tables, graphs, and figures*
How data were acquired	*Scanning electron microscopy (SEM); High-temperature universal testing machine.*
Data format	*Raw and analyzed*
Experimental factors	*Graphite infiltrated in fluoride molten salt for mechanical properties.*
Experimental features	*Compressive strength, tensile strength,* softening coefficient (*K*), *force-deformation curve, absorbed energy for fluoride molten salt infiltrated graphite determined on a High-temperature universal testing machine. SEM micrographs for fracture surfaces.*
Data source location	*Shanghai, China*
Data accessibility	*Data are with this article*
Related research article	*Can Zhang, Zhoutong He, Yantao Gao, et al. The effect of molten FLiNaK salt infiltration on the strength of graphite, J. Nucl. Mater. 512 (2018) 37–45*. https://doi.org/10.1016/j.jnucmat.2018.09.051[1]

**Value of the data**

• The systematic data include microstructural and mechanical properties for fluoride molten salt infiltrated graphite under different infiltration pressures and different graphite grades.
• Such data can be used to assess the compatibility of the graphite with fluoride molten salt at high temperature.
• The systematic properties can be used to predict the allowable stresses of molten salt infiltrated graphite, which can be used in safety analysis.

## 1 Data

**Fig. 1:**
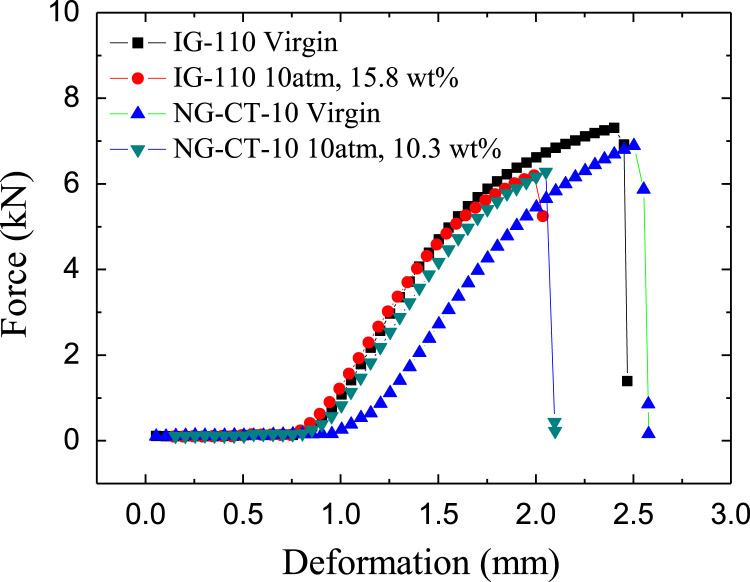
The force–deformation curves of graphite compression samples.

**Fig. 2:**
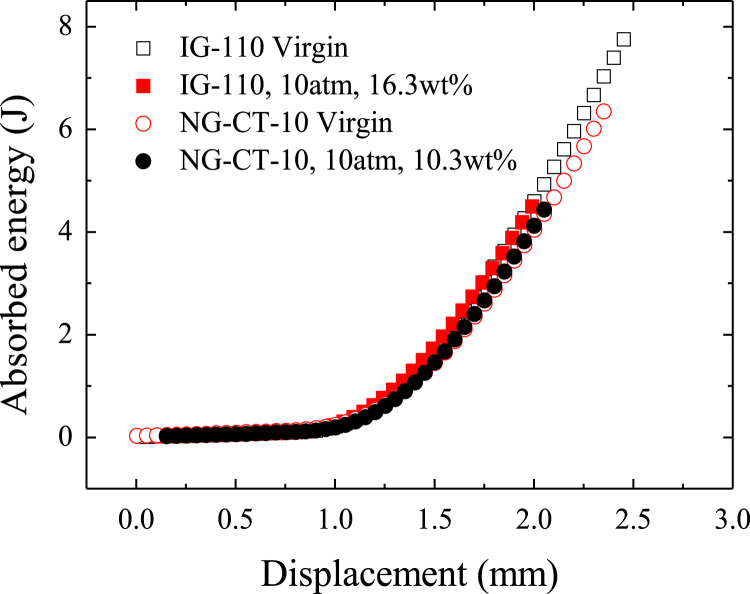
The absorbed energy during deformation and fracture process.

**Fig. 3:**
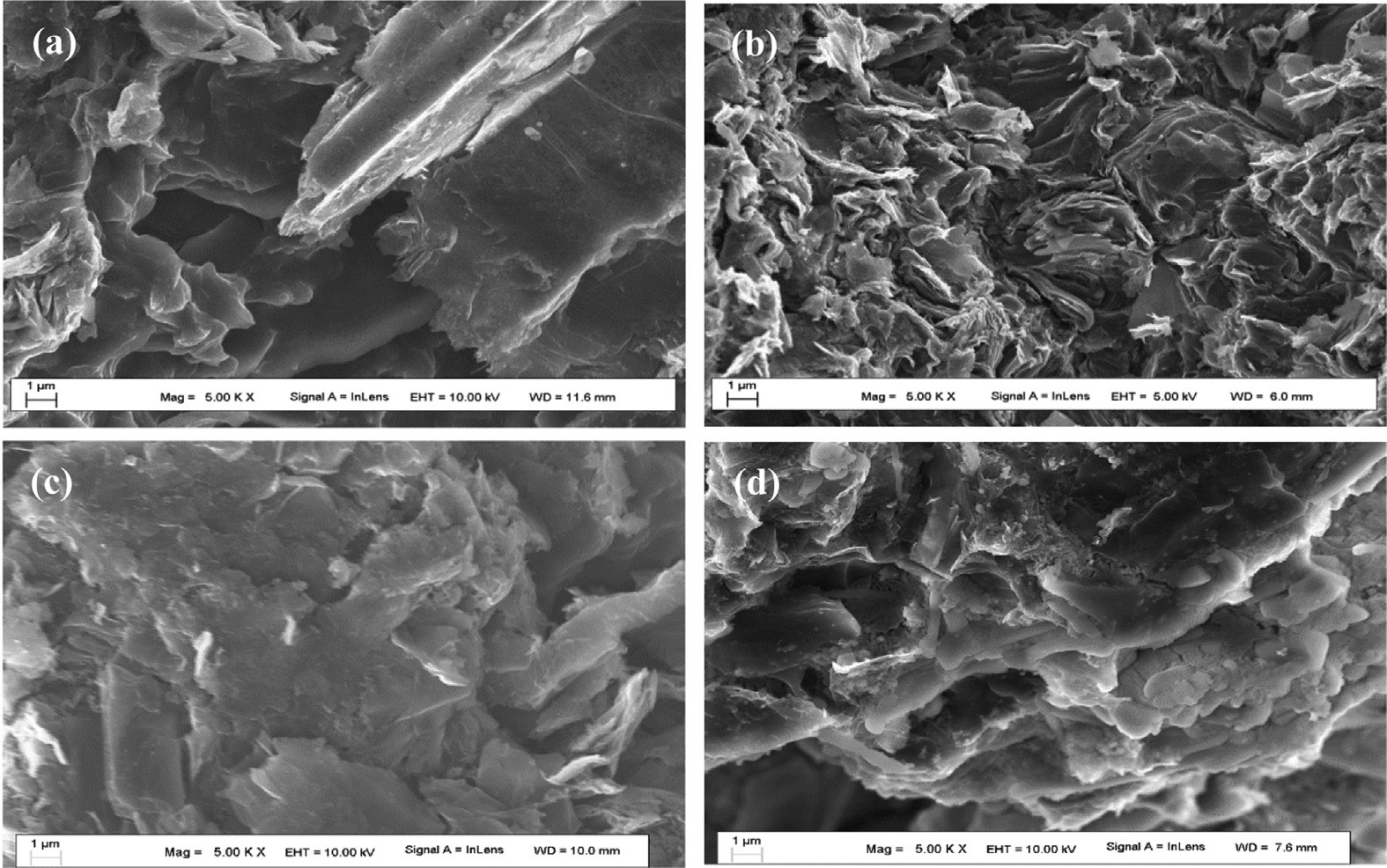
The SEM micrographs of the fracture surface of molten salt infiltrated graphite and virgin graphite. (a) and (b) The SEM images of virgin and 1000 kPa molten FLiNaK salt infiltrated NG-CT-10. (c) and (d) The SEM images of virgin and 1000 kPa infiltrated IG-110.

Systematic mechanical property and microstructural data of as-machined and molten FLiNaK salt infiltrated graphite at four different infiltration pressures (0 kPa, 450 kPa, 600 kPa, and 1000 kPa) are reported below. Included are the compressive strength, softening coefficient of compressive strength, tensile strength, softening coefficient of tensile strength, force-deformation curve, and absorption energy of fluoride molten salt infiltrated graphite at 700 °C (Table 1, Table 2, Table 3, Table 4, Figs. 1 and 2). Table 5 shows the diameter variation of graphite compression samples after 1000 kPa molten salt infiltration. Finally, Fig. 3 shows SEM micrographs of fracture surfaces for both molten salt infiltrated graphite and virgin graphite.

**Table 1:** The high-temperature compressive strength (mean±S.D.) under different infiltration pressures.

	IG-110		NG-CT-10	
Infiltration pressure (kPa)	Weight gain ratio (wt%)	Compressive strength (MPa)	Weight gain ratio (wt%)	Compressive strength (MPa)
0	0	93.4	0	90.3 ± 2.53
450	3.78 ± 0.87	83.9 ± 1.56	2.08 ± 1.27	86.4 ± 2.90
600	8.56 ± 1.05	74.8 ± 1.06	13.05 ± 0.21	84.1 ± 0.55
1000	9.90 ± 0.87	74.2 ± 2.71	15.60 ± 0.64	81.3 ± 0.99

**Table 2:** The softening coefficients of compressive strength (mean±S.D.) under different infiltration pressures.

Infiltration pressure (kPa)	*K*_*c*_ for IG-110	*K*_*c*_ for NG-CT-10
0	1	1
450	0.898 ± 0.017	0.957 ± 0.032
600	0.801 ± 0.011	0.931 ± 0.006
1000	0.794 ± 0.029	0.900 ± 0.011

**Table 3:** The high-temperature tensile strength (mean±S.D.) under different infiltration pressures.

	IG-110		NG-CT-10	
Infiltration pressure (kPa)	Weight gain ratio (wt%)	tensile strength (MPa)	Weight gain ratio (wt%)	tensile strength (MPa)
0	0	26.6 ± 0.9	0	23.6 ± 0.58
450	3.10 ± 0.01	25.7 ± 0.61	2.25 ± 0.01	23.1 ± 0.42
600	13.08 ± 0.47	25.2 ± 0.52	7.20 ± 0.51	21.8 ± 0.69
1000	14.30 ± 1.0	24.4 ± 0.87	9.73 ± 0.46	21.8 ± 0.33

**Table 4:** The softening coefficients of the tensile strength (mean±S.D.) under different infiltration pressures.

Infiltration Pressure (kPa)	*K*_*t*_ for IG-110 (N/A)	*K*_*t*_ for NG-CT-10 (N/A)
0	1	1
450	0.966 ± 0.023	0.975 ± 0.018
600	0.948 ± 0.020	0.923 ± 0.029
1000	0.916 ± 0.033	0.920 ± 0.014

**Table 5:** The diameter variation of graphite compression samples after 1000 kPa molten salt infiltration.

No.	Grade	Diameter (before) (mm)	Diameter (after) (mm)
1	IG-110	10.015	10.016
2	IG-110	10.021	10.020
3	IG-110	10.013	10.013
4	NG-CT-10	10.025	10.024
5	NG-CT-10	9.998	9.999
6	NG-CT-10	10.017	10.018
Average		10.015	10.015

## 2 Experimental design, materials and methods

### 2.1 Sample preparation

**Fig. 4:**
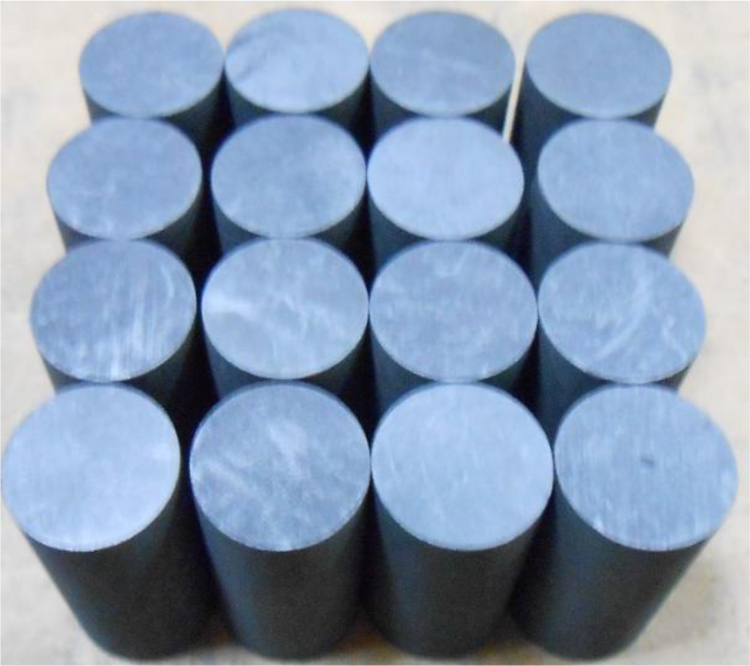
The picture of compression samples (NG-CT-10, *Φ*10.0 × 20.0 mm).

**Fig. 5:**
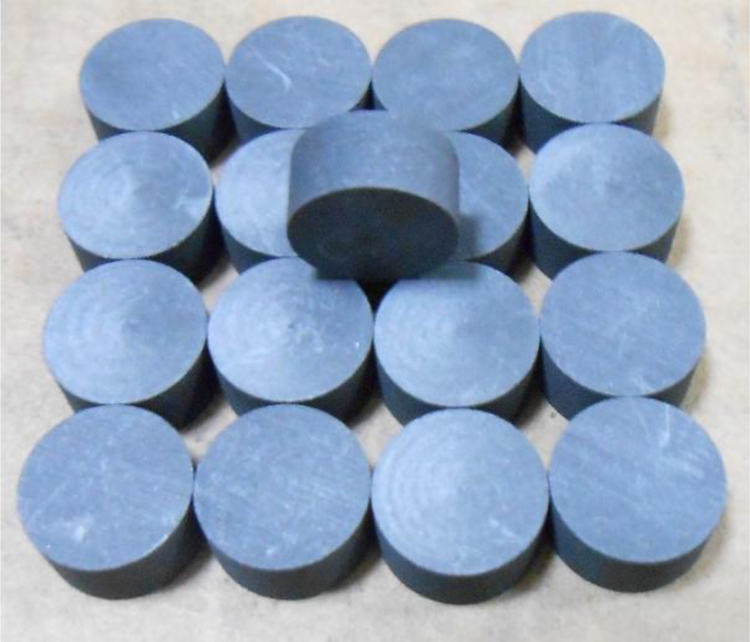
The picture of tension samples (NG-CT-10, *Φ*10.0 × 5.0 mm).

The graphite samples used in the experiment were NG-CT-10 from Chengdu Carbon Co., Ltd, and IG-110 from Toyo Tanso Co., Ltd. Graphite was machined to form the cylindrical samples with dimensions of *Φ*10.0 × 20.0 mm which were employed in compression tests, as shown in Fig. 4. Graphite was machined to form the disk specimens with dimensions of *Φ*10.0 × 5.0 mm which were employed in tension tests, as shown in Fig. 5. Before molten salt infiltration, the samples were marked by a laser marking machine, cleaned with ethyl alcohol and vacuum dried for 2 h at 150 °C.

### 2.2 Graphite samples infiltration in molten salt

**Fig. 6:**
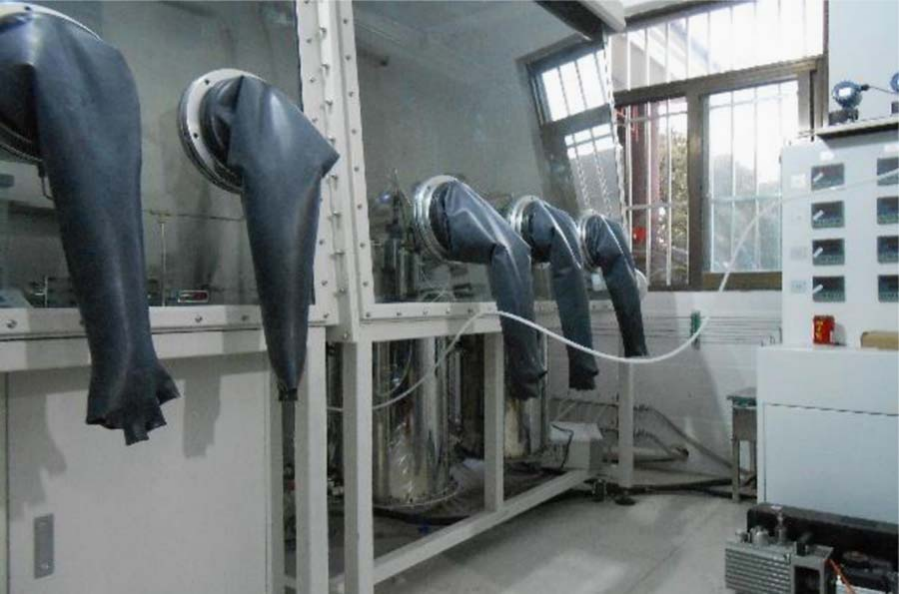
Glove box and pressure vessels.

**Fig. 7:**
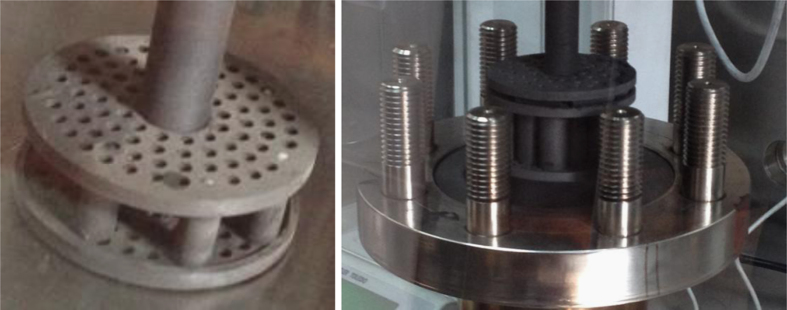
Infiltration equipment.

After the initial weights and the dimensions were recorded, five graphite samples were infiltrated in a pressure vessel at 700 °C using approximately 0.5 L salt for times of 20 h. The molten salt used was the eutectic salt of LiF, NaF, and KF (FLiNaK, LiF-NaF-KF: 46.5-11.5-42 mol%) with melting point ~450 °C. The graphite samples were degassed before being immersed in molten salt. Infiltration pressure used includes 0 kPa, 450 kPa, 600 kPa, and 1000 kPa in the experimental. The infiltration equipment was shown in Figs. 6 and 7. After the graphite samples were removed from the molten salt after infiltration experiment, weight change and dimensional change of the samples were determined by weighting graphite and measuring the dimensions in a dry (H_2_O ≤ 1 ppm), low oxygen (O_2_ ≤ 1 ppm) argon atmosphere glove box.

### 2.3 Mechanical testing at high temperature

**Fig. 8:**
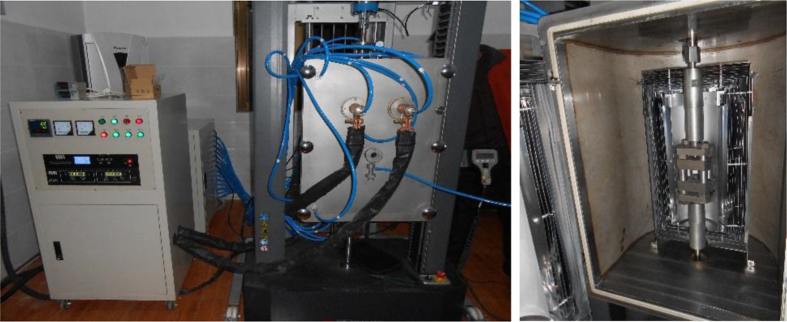
High-temperature universal testing machine.

The mechanical testing was carried out at a high-temperature universal testing machine (HTUTM), which was composed of a sealed high-temperature chamber and a universal testing machine, as shown in Fig. 8. There is a door in the chamber, through which the specimens can be changed.

The samples were heated to 700 °C at a rate of ~10 °C/min and maintained about 1 h thereafter. Then, compression tests with a constant crosshead rate of 1.2 mm/min were performed. And the tension tests with a constant loading force rate of 6 N/s was carried out. During the compression process, the absorption energy (*E*_*a*_) of the samples during deformation and fracture process will be evaluated by the integration of force to displacement (Eq. (1)) [2]

(1) $$E_{a}\approx \int_{L}FdL$$

where *L* is displacement of crosshead, and *F* is the force during a test. The fracture fragments of compression tests were measured using scanning electron microscopy (SEM; LEO 1530VP).
