# Supplementary material for: Dataset on the mechanical property of graphite after molten FLiNaK salt infiltration
Source: Data Brief. 2018 Nov 14;21:1963–9. doi: 10.1016/j.dib.2018.11.036 (PMC6258876; doi:10.1016/j.dib.2018.11.036)
Supplement: Supplementary file 1 — Supplementary material. [file mmc1.docx]

**Conflict of Interest Form**

We confirm that the manuscript has been read and approved by all named authors and that there are no other persons who satisfied the criteria for authorship but are not listed. We further confirm that the order of authors listed in the manuscript has been approved by all of us.

Thank you and best regards.

Sincerely yours,

Can Zhang

**Email: zhangcan@sinap.ac.cn**
